# Human health risks from heavy metals in fish of Buriganga river, Bangladesh

**DOI:** 10.1186/s40064-016-3357-0

**Published:** 2016-10-03

**Authors:** Md. Kawser Ahmed, Mohammad Abdul Baki, Goutam Kumar Kundu, Md. Saiful Islam, Md. Monirul Islam, Md. Muzammel Hossain

**Affiliations:** 1 Department of Oceanography, University of Dhaka, Dhaka, 1000 Bangladesh; 2 Department of Zoology, Jagannath University, Dhaka, 1000 Bangladesh; 3 Department of Fisheries, University of Dhaka, Dhaka, 1100 Bangladesh; 4 Department of Risk Management and Environmental Sciences, Graduate School of Environment and Information Sciences, Yokohama National University, Yokohama, Kanagawa 240-8501 Japan

**Keywords:** Heavy metal, Fish, Human health, Target hazard quotient (THQ), Hazard index (HI), Buriganga river, Bangladesh

## Abstract

Heavy metals are known to cause deleterious effects on human health through food chain. Human health risks were evaluated from consumption of heavy metal contaminated fish from Buriganga River in Bangladesh. Whole body of five fish species (*Puntius ticto, Puntius sophore, Puntius chola, Labeo rohita* and *Glossogobius giuris*) were analyzed which contained various concentrations of Cd, As, Pb, Cr, Ni, Zn, Se, Cu, Mo, Mn, Sb, Ba, V and Ag. Concentrations of Mn, Zn, Se and Pb in all fish species were above the Food Safety Guideline (FSG) by WHO/FAO. Assessment of noncarcinogenic health hazard by target hazard quotient (THQ) indicated no concern from consumption of these fish except for Mn. However, all metals together may affect human health as revealed by hazard index (HI). The target cancer risk (TR) values suggested carcinogenic risk from Ni and As. Taken together it can be concluded that there is potential human health risk in consuming fish from river Buriganga.

## Background

Chemical contamination of food is considered of the most significant sources of human health risks. The most significant sources of foodborne diseases are microbiological and chemical hazards. Health risk due to consumption of food from aquatic ecosystems contaminated with hazardous chemicals including metals has increased globally especially in developing countries such as Bangladesh. The increasing usage of heavy metals in industry has led to increased release of harmful heavy metals into the aquatic environment (Agusa et al. 2005, 2007; Hajeb et al. 2009). The increasing demand of food safety research has accelerated regarding the risk associated with consumption of food contaminated by toxic metals (Mansour et al. 2009; Saha and Zaman 2013).

Metals and metalloids from natural and anthropogenic sources continuously enter the aquatic environment where they pose a serious threat to human and ecological health, owing to their toxicity, long persistence, bioaccumulation, and biomagnifications in the food chain (Rahman et al. 2013). Essential metals (Cu, Co, Zn, Fe, Ca, Mg, Se, Ni and Mn) are required in very trace quantities for the proper functioning of enzyme systems, hemoglobin formation and vitamin synthesis in human. However, excess of these essential metals results in metabolic disturbances (Hina et al. 2011). Pb, Ba, Cd, Hg, Cr, and As have no established role in biological system (Canli and Atli 2003) have been identified as toxic heavy metals and their maximum recommended residual levels have been set for humans (FAO 1983; European Commission 2006; FDA 2001). Heavy metals accumulate in vital organs in the human body such as the kidneys, bones, and liver which result in numerous serious health effects such as neurotoxic and carcinogenic effects (Duruibe et al. 2007; Sapkota et al. 2008). Among various heavy metals, Cr and Ni are known to cause various pulmonary disorders (Forti et al. 2011), while high intake of Cu can cause liver and kidney damage (Tuzen 2009; WHO 1995). Cd is toxic to the cardiovascular system, kidneys, and bones (Fang et al. 2014); excessive intake of Zn negative effects on immunological system (reduction in lymphocyte stimulation response) and cholesterol metabolism (Goldhaber 2003).

Fish assimilate metals by ingestion of particulate material suspended in water, ingestion of food, ion-exchange across lipophilic membranes (e.g., the gills), and adsorption on tissue and membrane surfaces. As a result, fish may accumulate large amounts of metals from the water (Mansour and Sidky 2002) and the accumulation rate is dependent both on uptake and elimination rates (Guven et al. 1999). So, there have been several studies and monitoring programs worldwide on metal accumulation in fish (Erdoĝrul and Ates 2006; Rashed 2001). Fish is known as integral component of a well-balanced diet worldwide because it provides energy, proteins, vitamins and different nutrients also (Pieniak et al. 2010). Studies have reported that dietary intake is the main route of exposure to heavy metals for most people (Zhuang et al. 2009). Fish have been reported in several studies as a source of heavy metals in human through consumption (Castro-González and Méndez-Armenta 2008). That’s why fish has been considered as one of the most indicative factors in freshwater systems, for the estimation of trace metals pollution and risk potential of human consumption (Alhashemi et al. 2012; Pan and Wang 2012; Yi et al. 2011; Vieira et al. 2011).

In Bangladesh, about 900 industries dispose untreated industrial wastes directly into rivers. The Buriganga River is the most polluted river in Bangladesh (Nouri et al. 2009) which is being increasingly polluted with huge volumes of toxic wastes from thousands of industrial units and sewerage lines (Islam et al. 2006). However, a wide variety of heavy metals originate from industrial waste discharge, batteries, lead based paint and gasoline discharge from cargos, launch and mechanized boat, traffic and improper domestic waste discharge etc. Although there has been several studies reporting enrichment of heavy metals in water, sediment and fish in various rivers including Buriganga (Begum et al. 2013; Ahmed et al. 2010b; Mohiuddin et al. 2011; Saha and Hossain 2011) studies reporting the risk assessment with special focus on human health are scant. But the risk assessment of these metals via daily dietary intake is a very important issue (Martí-Cid et al. 2008). Only Ahmed et al. (2015) and Islam et al. (2015) carried the carcinogenic and non-carcinogenic risk of several heavy metals in three fish and one prawn species collected from Buriganga. However, the accumulation and magnification vary in different fish species. In the context, it is important to monitor the concentration and potential human health risk associated with consumption of commonly consumed fish species. So, this study aims to determine various trace metals in fish tissue and to compare the estimated intakes with references toxicological and nutritional values. This study also evaluates the carcinogenic and non-carcinogenic health risk for humans through fish consumption.

## Methods

### Collection of samples

**Fig. 1:**
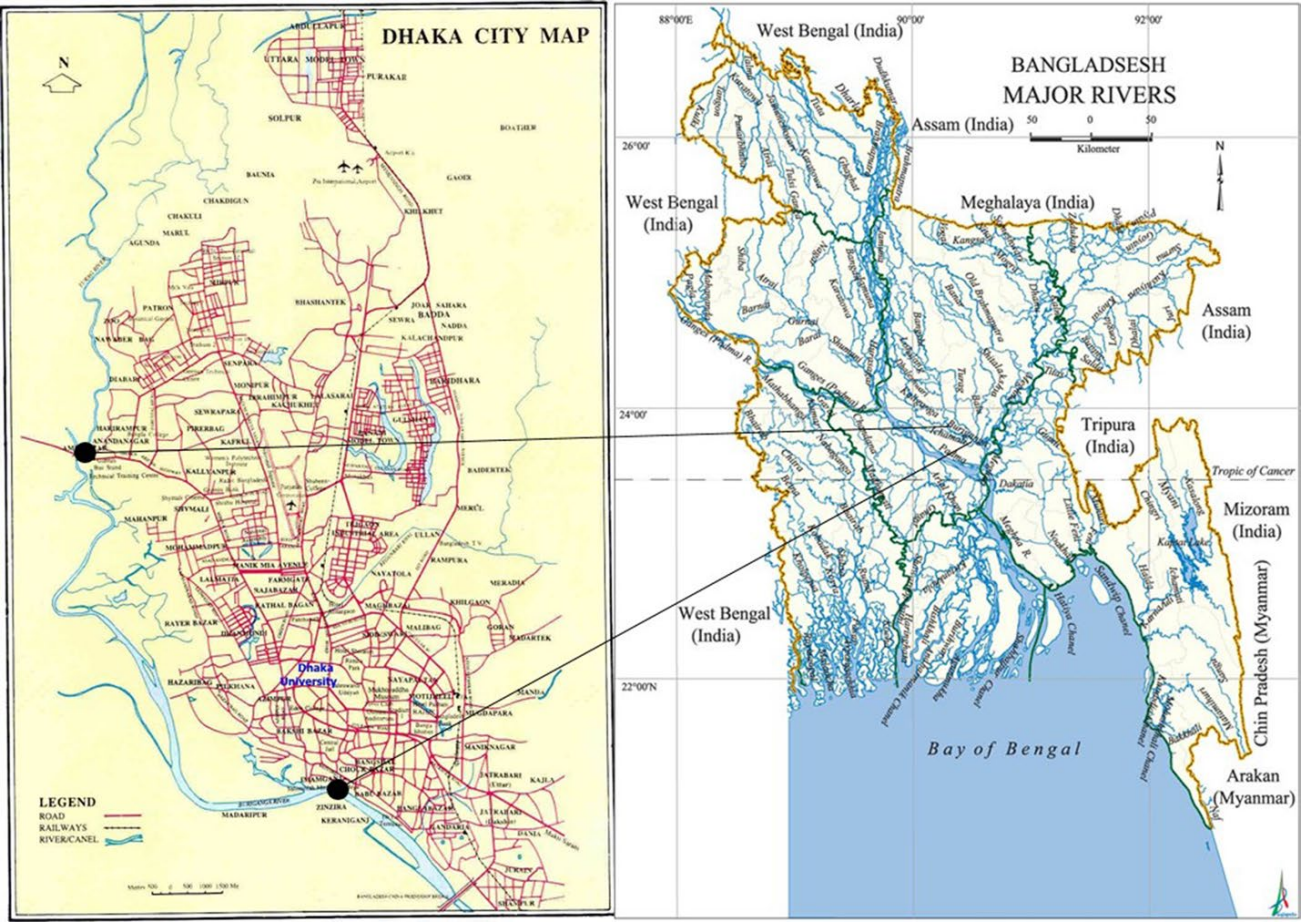
Location of Buriganga river and the sampling area (between* two dots*)

Fish samples were collected from different stations (Fig. 1) of the river Buriganga near Kamrangir Char in between the Bangladesh–China friendship bridge-01 (23°40′N 90°20′E) and Amin Bazzar Bridge (23°47′N 90°20′E) where fishing effort is high. Fishes were collected from the professional fishermen while they were fishing in the river during August to September, 2013. Individuals of the same species were of similar size and weight. The samples were immediately preserved in air sealed plastic bags and transported to the laboratory. Five fish species were identified namely: *Puntius ticto, Puntius sophore, Puntius chola, Labeo rohita* and *Glossogobius giuris* and their mean weight were 2.00 g, 2.40 g, 1.30 g, 47.18 g, and 5.44 g respectively.

### Preparations of samples

Whole body of fish was used for analyses following Islam et al. (2015). A composite sample for each species was prepared and homogenized in a stainless steel blender cup and 50 g test portions were stored at −20 °C. The samples were then freeze dried and sealed in airtight plastic bags. The dried samples were transported to Yokohama National University, Japan for analytical methods.

### Analytical methods

#### Sample digestion

0.2 g of dried fish sample were digested in DAP-Three step-digestion procedure with 5 ml HNO_3_ (Kanto Chemical Co, Japan) and 2 ml H_2_O_2_ (Wako Chemical Co, Japan) in microwave digestion system (Berghof Microwave MWS-2, Germany) as described by Islam et al. (2015). Three replicas were prepared for each sample was during digestion. Samples were then transferred into a Teflon beaker and total volume was made up to 50 ml with MilliQ water (Elix UV5 and MilliQ, Millipore, USA) and filtered with PTTF syringe filter (pore size = 0.45 mm) and stored in crew cap plastic tube.

#### Instrumental analysis and quality assurance

The samples were analyzed for cadmium (Cd), arsenic (As), lead (Pb), chromium (Cr), nickel (Ni), zinc (Zn), selenium (Se), copper (Cu), molybdenum (Mo), manganese (Mn), antimony (Sb), barium (Ba), vanadium (V), silver (Ag) using ICP-MS (Agilent 7700, USA). For each run of analysis blank, certified reference materials (CRM) as internal standard along with the samples were analyzed in duplicate to eliminate any batch-specific error. Multi-element standard solution was used to prepare standard curve. Five standards with standard linear regression and internal standardization were prepared at levels ranging from 0–50 µg/L. All test batches were evaluated using an internal quality approach and validated if they satisfied the defined internal quality controls (IQCs).

### Non-carcinogenic Health hazard and carcinogenic risk estimation

The target hazard quotient (THQ) assessed the non-carcinogenic health hazards for each individual metal through fish consumption, while the hazard index (HI) was estimated as the sum of the THQs (USEPA 2011). The THQ assumes a level of exposure (i.e., RfD) below which it is unlikely for even sensitive populations to experience adverse health effects. On the other hand HI indicates the combined hazard of all metals.

For carcinogens, Target risks (TR) were estimated as the incremental probability of an individual to develop cancer over a lifetime, as a result of exposure to that potential carcinogen (i.e., incremental or excess individual lifetime cancer risk (USEPA 1989). The THQ, HI and TR values were estimated using Eqs. (1), (3), and (4) respectively. European Protection Agency (EPA) had declined RfD value for Pb (USEPA 2004) So, the THQ for Pb was calculated using the Eq. (2) (Liu et al. 1996):

1 $$THQ = \frac{EF \times ED \times FIR \times Cf \times CM}{WAB \times ATn \times RfD} \times 10^{ - 3}$$

or,

2 $$THQ = \frac{CM}{MRL}$$

3 $$\begin{aligned} {\text{HI}} &= \,{\text{THQ}}\left( {\text{V}} \right)+{\text{ THQ}}\left( {\text{Cr}} \right)+{\text{ THQ}}\left( {\text{Mn}} \right)+{\text{ THQ}}\left( {\text{Ni}} \right)+{\text{ THQ}}\left( {\text{Cu}} \right) \\ &\quad+{\text{ THQ}}\left( {\text{Zn}} \right)+{\text{ THQ}}\left( {\text{As}} \right) + {\text{ THQ}}\left( {\text{Se}} \right)+{\text{ THQ}}\left( {\text{Mo}} \right)+{\text{ THQ}}\left( {\text{Ag}} \right)\\ &\quad+{\text{ THQ}}\left( {\text{Cd}} \right)+{\text{ THQ}}\left( {\text{Sb}} \right)+{\text{ THQ}}\left( {\text{Ba}} \right)+{\text{ THQ}}\left( {\text{Pb}} \right) \end{aligned}$$

4 $$TR = \frac{EF \times ED \times FIR \times CF \times CM \times CPSo}{WAB \times TAc} \times 10^{ - 3}$$

where, THQ is the target hazard quotient;* EF* is the exposure frequency (365 days/year);* ED* is the exposure duration (30 year for noncancer risk as used by USEPA 2011),* FIR* is the fish ingestion rate (49.41 g/person/day; (BBS 2014);* Cf* is the conversion factor (00.208) to convert fresh weight (F_W_) to dry weight (D_w_.) considering 79 % of moisture content in fish; *CM* is the heavy metal concentration in fish (mg/kg d.w.); *WAB* is the average body weight (bw) (70 kg); *ATn* is the average exposure time for noncarcinogens (*EF* × *ED*) (365 days/year for 30 year (i.e., ATn = 10,950 days) as used in characterizing noncancer risk (USEPA 2011); RfD is the reference dose of the metal according to the Human health risk assessment. Regional screening level (RSL) summary table (USEPA 2015);* MRL* is maximum regulation limit for muscle meat for fish set by the Regulation (EC) No 1881/2006 (European Commission 2006).* CPSo* is the carcinogenic potency slope, oral (mg/kg bw-day-1);* ATc* is the averaging time, carcinogens (365 days/year for 70 year as used by (USEPA 2011). Since* CPSo* values were known for Ni, AS, Cd and Pb, so* TR* values were calculated for intake of these metals.

## Results and discussion

### Metal concentration in fish species

Fish muscle is the main part of human diet for most species throughout the world. Except *L. rohita* other fish species in this study were small indigenous species (SIS) which are generally consumed whole by Bangladeshi population. So, the whole body of fish was analyzed for different metals. All fish species contained V, Cr, Mn, Ni, Cu, Zn, As, Se, Mo, Ag, Cd, Sb, Ba, and Pb at different concentrations (Mean ± SD) (Table 1). Zn was found at the highest concentration in all fish species followed by Mn, Cu and Cr. The trend was Zn > Mn > Ba > Cu > Cr > Pb > V > Ni > Se > As > Cd in almost all fish species. Similar trend was also reported in *L. rohita* by Javed and Usman (2011) Earlier studies also reported Zn to be at highest concentration in fish collected from Buriganga river (Begum et al. 2013; Ahmed et al. 2010b, 2015; Khan et al. 2014 ). Concentration of various metals in fish usually follow Fe > Zn > Pb > Cu > Cd (Jezierska and Witeska 2006) which is the similar to the findings of the present study. However, the magnitude of bioaccumulation is a function of species and trophic transfer (Spry and Wiener 1991). So, species at different positions in the food chain accumulate different concentrations of metals. In the present study, there were considerable variations in the concentrations of heavy metals among different species. Among the five fish species *L. rohita* contained the highest concentrations of almost all metals (Table 1). This was due to the larger size individuals of the species because larger fish tend to accumulate more heavy metals (Farkas et al. 2003). This phenomenon might also be the due to extensive column feeding nature of *L. rohita*. In contrast, *G. giuris* was found to have the lowest metal accumulation due to the smaller body size which reduces the metal accumulation through surface action. The findings of Ahmed et al. (2010b) in *G. giuris* were very close to this study. Metal accumulation in *P. sophore* and *L. rohita* in the present study were different from the concentrations reported in these species from other rivers (Ahmed et al. 2010a; Hasan et al. 2011). This is probably due to the variation in the concentrations of metal in the ambient water along with the variation in the size and age of the fish under investigation. In addition, metal speciation in the aquatic system as well as the pH and temperature are also the factors of metal accumulation (Moiseenko and Kudryavtseva 2001; Dhanakumar et al. 2015). Although permissible limits of heavy metals in fish and food have been recommended by various international and regional bodies, but no such value were reported for V, Mo, Ag and Ba. However, permissible limits have not been recommended yet to all heavy metals. Concentrations of Mn, Zn, Se and Pb were higher than the Food safety guidelines (FSG) in fish recommended by various parties (Table 1) making these fish species from Buriganga unacceptable as food. No such standard for V, Mo, Ag and Ba were found.

**Table 1:** Mean (± SD) heavy metals concentration (mg/kg ww) in five fish species collected from Buriganga river, Bangladesh

Heavy metals	Concentrations of metals (mg/kg ww) in different species					FSG (mg/kg ww)	Reference
	*Puntius ticto*	*Puntius sophore*	*Puntius chola*	*Labeo rohita*	*Glossogobius giuris*		
V	2.02 ± 0.15	1.36 ± 0.10	1.30 ± 0.08	5.72 ± 0.23	1.43 ± 0.83	Not reported	–
Cr	5.54 ± 1.52	4.33 ± 1.35	3.57 ± 1.60	18.84 ± 1.72	5.13 ± 0.96	12.0–13.0	USFDA (1993)
Mn	34.98 ± 1.01	22.42 ± 0.70	29.09 ± 0.91	125.81 ± 2.57	27.89 ± 1.69	1.0	FAO (1983)
Ni	1.65 ± 0.27	1.21 ± 0.30	1.00 ± 0.52	6.64 ± 0.24	0.73 ± 0.19	80.0	USFDA (1993)
Cu	11.52 ± 3.34	9.04 ± 1.57	6.86 ± 1.11	18.77 ± 2.18	5.90 ± 0.50	30.0	WHO (1995)
Zn	203.58 ± 12.85	248.20 ± 14.63	292.13 ± 19.75	251.69 ± 18.17	194.68 ± 12.57	30.0	FAO (1983)
As	0.32 ± 0.01	0.19 ± 0.01	0.17 ± 0.00	0.73 ± 0.03	0.20 ± 0.01	1.0	FAO/WHO (2002)
Se	1.97 ± 0.58	1.68 ± 0.59	1.76 ± 0.68	1.81 ± 0.46	1.98 ± 0.14	1.0	MHSAC (2005)
Mo	0.08 ± 0.00	0.18 ± 0.00	0.06 ± 0.00	0.17 ± 0.01	0.05 ± 0.00	Not reported	–
Ag	0.12 ± 0.04	0.04 ± 0.01	0.02 ± 0.00	0.01 ± 0.00	0.01 ± 0.00	Not reported	–
Cd	0.02 ± 0.00	0.02 ± 0.00	0.01 ± 0.00	0.04 ± 0.00	0.01 ± 0.00	0.5 0.05	FAO (1983) EC (2006)
Sb	0.03 ± 0.00	0.02 ± 0.00	0.02 ± 0.00	0.06 ± 0.00	0.02 ± 0.00	1.0	FAO/WHO (2002)
Ba	17.92 ± 1.15	16.53 ± 0.88	23.06 ± 1.30	19.18 ± 1.01	7.31 ± 0.33	12 mg/day	SCHER (2012)
Pb	3.05 ± 0.09	3.16 ± 0.08	2.32 ± 0.08	6.98 ± 0.23	1.77 ± 0.10	2.0 1.5	WHO (1995); EC (2006)

V is an essential element for normal cell growth and essential component of some enzymes, particularly the vanadium-nitrogenase used by some nitrogen fixing microorganisms. Although V is toxic when present at higher concentrations, the V complexes can reduce growth of cancer cells and improve human diabetes mellitus. However, V has not been classified by the United State Environmental Protection Agency (USEPA) or World Health Organization (WHO) to have a permissible level. The estimated daily upper boundary range of vanadium is 0.2 mg/day (USEPA 2011). There is no maximum level established for dietary intake of V through fish consumption in Bangladesh.

The Cr concentrations in this study were higher than that of reported by Ahmed et al. (2010b) This increasing trend might have resulted from the effluent coming from the tannery industries near the Buriganga river. Metal accumulation in the body of organisms increase with time of exposure until it reaches the equilibrium with the environment based on the partitioning coefficient of the chemical. Several studies have shown that chromium (VI) compounds can increase the risk of lung cancer (Ishikawa et al. 1994). Fish health can also be affected by Cr exposure. The presence of Cr along with other metals was reported to increase the glycogen level in different organs indicating the stress due to the metal exposure (Javed and Usmani 2011). Equivalent concentration of Cr found in Buriganga river were reported to cause DNA damage and also induce micronucleus in air breathing catfish (Ahmed et al. 2013).

Mn occurs naturally and may be released into water bodies through runoff or leaching facilitated by agricultural activities while anthropogenic sources include agro chemicals. Mn concentrations in the present study were the highest among all metals. According to previous literature, the Mn concentrations in the samples ranged from 0.54 mg/kg to 79.08 mg/kg (Bashir et al. 2013); the muscles of fishes found in Indian fish markets ranged from 0.14 mg/kg to 3.36 mg/kg (Sivaperumal et al. 2007). Mn is an essential element in human and Mn deficiency causes skeletal and reproductive abnormalities (Sivaperumal et al. 2007). However, excess intake of Mn can result in psychological and neurologic disorder (Moreno et al. 2009).

Ni measured relatively low concentrations in the samples compared to Cr and Mn. The concentrations of Ni in the samples ranged between 0.73 ± 0.19 and 6.64 ± 0.24 mg/kg. Similar kinds of results were also observed in Cu, Zn, and Pb concentrations.

Zn is an essential micronutrient for all organisms. Zn is required at high level in organism for maintaining certain biological function as a constituent of various enzymes. Zn was found in very high concentrations in all fish species in the present study which exceeded the guideline values of FAO (1983). Both Zn and Mn were reported in *Mastacembelus armatus* at higher concentrations than FAO/WHO recommended levels in an effluent dominated rivulet in India (Javed and Usmani 2013).

As in natural surface water is less available settles down to sediment along with Fe and become unavailable to fish for uptake. A sublethal concentration of As can pose mutation or DNA damage to fish (Ahmed et al. 2011) Arsenic is present in our food in different chemical forms. The toxic inorganic arsenic becomes organic through methylation in aquatic environment which is less toxic. In the present study, the total arsenic concentrations were estimated. Although inorganic arsenic was estimated to be 10 % of total arsenic (USFDA 1993) but it is difficult to conclude about the contribution of different the forms of arsenic in total concentration. Chronic exposure to inorganic arsenic may cause several health effects, including to the gastrointestinal tract, respiratory tract, skin, liver, cardiovascular system, hematopoietic system and the nervous system (Mandal and Suzuki 2002).

Se is an essential micronutrient but at high concentrations it can be toxic (Coyle et al. 1993). About 1 mg/kg (ww) of Se in prey is the threshold for toxicity in some fish, of 2.6 mg/kg (ww) in muscle is associated with adverse in the fish themselves (Lemly 1993a, b).

Sb and Cd were present at a concentration lower than WHO/FAO standards (Table 1). This was due to the source of Sb and Cd is not significantly contributing the heavy metal load in Buriganga. Cd is a serious contaminant, a highly toxic element, which is transported in the air. Cd concentrations in all fish species were well below the maximum permissible limit in fish by FAO and European Commission (Table 1). Ahmed et al. (2010a) investigated the heavy metal concentration in fish and oyster from the Shitalakhya River, Bangladesh and found seasonal variation in Cd concentration ranged from 1.09 to 1.21 mg/kg. Industrial processes such as smelting or electroplating and the fertilizers can contribute to the environmental concentration of Cd. to Cd was reported to cause kidney failure and softening of bones following long-term or high dose exposure (Vannoort and Thomson 2006), and high levels of Cd have been reported to prostate cancer (Gray et al. 2005).

In the present study the highest concentration of Ba was 23.06 ± 1.30 (Table 1). Small amounts of water-soluble Ba may cause a person to experience breathing difficulties, increased blood pressures, heart rhythm changes, stomach irritation, muscle weakness, changes in nerve reflexes, swelling of brains and liver, kidney and heart damage. The maximum tolerable daily intake recommended by the Scientific Committee on Health and Environmental Risks as 12 mg/day (SCHER 2012). Pb is a ubiquitous pollutant which could find its way into the Buriganga River through discharge of industrial effluents from various industries such as printing, dyeing, oil refineries, textile around Dhaka City, and other sources.

### Non-carcinogenic health hazard and carcinogenic risk

The health risk assessments are based on assumptions that for most chemicals with noncancer effects, exhibit a threshold response. The Target hazard quotient (THQ) estimated for individual heavy metals through consumption of different fish species are presented in Table 2. The acceptable guideline value for THQ is 1 (USEPA 2011). THQ values were less than 1 for all individual heavy metal except Mn in *L. rohita* (3.694) and *P. ticto* (1.027) indicating no potential non-carcinogenic health risk from ingestion of a single heavy metal through consumption of these fishes. In contrast, the combined impacts of all metals under consideration were higher than the acceptable limit of 1 for HI in all species. Consumption of *L. rohita* is of biggest concern with a HI value over 5 (Table 2). Humans are often exposed to more than one pollutant and suffer combined or interactive effects (Li et al. 2013). *L. rohita* was with the highest hazard potential, while there was no significant difference in HI among other fish species (Table 2). But the HI might overestimate the potential for noncancer health effects. This is because the toxicological effects associated with exposure to multiple chemicals, often through different exposure pathways, may not be additive. Moreover, the effect of one metal is supposed to be dependent on the others due to the competitive absorption of metal ions in specific tissues of concern. The risk associated with the carcinogenic effects of target metal is expressed as the excess probability of contracting cancer over a lifetime of 70 years. However, the THQ and HI are not direct measurement of risks because it does not define any dose–response relationship (USEPA 1989).

**Table 2:** Target hazard quotient (THQ) for different heavy metals and their hazard index (HI) from consumption of five fish species collected from Buriganga River, Bangladesh

THQ					
Heavy metals	*Puntius ticto*	*Puntius sophore*	*Puntius chola*	*Labeo rohita*	*Glossogobius giuris*
V	0.059	0.040	0.038	0.168	0.042
Cr	0.271	0.212	0.175	0.922	0.251
Mn	1.027	0.658	0.854	3.694	0.819
Ni	0.024	0.018	0.015	0.097	0.011
Cu	0.042	0.033	0.025	0.069	0.022
Zn	0.100	0.121	0.143	0.123	0.095
As	0.157	0.093	0.083	0.357	0.098
Se	0.058	0.049	0.052	0.053	0.058
Mo	0.002	0.005	0.002	0.005	0.001
Ag	0.004	0.001	0.001	0.000	0.000
Cd	0.003	0.003	0.001	0.006	0.001
Sb	0.011	0.007	0.007	0.022	0.007
Ba	0.013	0.012	0.017	0.014	0.005
Pb	0.001	0.002	0.001	0.003	0.001
**HI**	1.773	1.255	1.414	5.535	1.412

Epidemiological studies have shown that Ni and Cd correlate with increased incidences of cancer in humans (Nordberg 2010). Ni, As and Cd belong to group 1 of the International Agency for Research on Cancer classification system (IARC 2014) with sufficient evidence of carcinogenicity in human. The TR values were estimated for the metals reported with known carcinogenic effects. The TR values for Ni, As, Cd and Pb are presented in Table 3. In general, the TR values lower than E−6 are considered to be negligible for carcinogenic risk, cancer risks above E−04 are considered unacceptable (USEPA 1989, 2010) and risks lying between E−6 and E−4 are generally considered an acceptable range (Fryer et al. 2006). The present study found that all five fish species poses carcinogenic risk from Ni consumption, while only *L. rohita* had risk from As. Ahmed et al. (2015) reported similar result for some other fish species collected form river Buriganga.

**Table 3:** Target Cancer Risk (TR) of heavy metals from consumption of five fish species collected from Buriganga River, Bangladesh

TR					
Heavy metals	*Puntius ticto*	*Puntius sophore*	*Puntius chola*	*Labeo rohita*	*Glossogobius giuris*
Ni	4.13E−04	3.03E−04	2.50E−04	1.66E−03	1.83E−04
As	7.1E−05	4.2E−05	3.8E−05	1.61E−04	4.4E−05
Cd	1.9E−05	1.9E−05	9.3E−06	3.7E−05	9.3E−06
Pb	3.8E−06	4E−06	2.9E−06	8.7E−06	2.2E−06

## Conclusions

The present study concludes that the commonly consumed fish species collected from river Buriganga contained various concentrations of heavy metals and the degree of accumulation vary among different species. Cr, Mn, Ni, Cu, Zn, Pb were present at higher concentrations than the Maximum allowable concentrations (MAC) in fish recommended by FAO/WHO. The metals do not pose non-carcinogenic health hazard individually but their combined effect is potentially hazardous to human health. The accumulation of Ni in all fish species suggests significant cancer risk through consumption of these fish species.
